# Variola, or Small-Pox

**Published:** 1838-06-20

**Authors:** N. Chapman

**Affiliations:** Professor of the Theory and Practice of Physic, in the University of Pennsylvania


### THE MEDICAL EXAMINER.

DEVOTED TO MEDICINE, SURGERY, AND THE COLLATERAL SCIENCES.

EDITED BY J. B. BIDDLE, M. D. AND M. CLYMER, M. D.

No. 13.] PHILADELPHIA, WEDNESDAY, JUNE 20, 1838. [Vol. I.

### VARIOLA, OR SMALL-POX.

*By* N. Chapman, M. D., *Professor of the Theory and Practice of Physic, in the University of Pennsylvania.*

(Continued from page 185.)

Diversified, as this disease is, by gradations of severity, as well as adventitious complications, the phenomena, on dissection, must, of course, be materially different. Generally, the mucous membrane of the stomach and small intestines, exhibits simple phlogosis, is florid and highly injected, in patches, or more diffused, and that of the lungs often in a similar condition, though usually not so intense, with the brain, particularly its arachnoid tissue, scarcely less affected. But, in very violent attacks, the ravages of the disease are manifested throughout the structure of the parts enumerated, to the destruction of organization, in which condition is also sometimes found most of the contents of the abdomen.

The case, however, being of a typhoid, or congestive character, the phenomena, of course, are correspondently modified. Connected with pervading lesions of the mucous membranes, such as livid or ecchymosed blotches, and, sometimes, softenings, erosions, or ulcerations, there are venous congestions of the solid organs, in substance, and investing tissues, with effusions, more or less, in the great cavities. Most of the old writers represent the state of things as that of one mass of putrefaction.

Great attention, of late, has been paid, both in France and England, to *post mortem* examinations, in this disease. We learn, that, in the "Maison des Enfans," at Paris, "the appearances were, inflammation and ulceration of the internal coat of the intestines, with pustular eruptions there, and frequently more or less of peritonitis. In three cases, a false membrane, as in croup, was also discovered, lining the whole alimentary canal, from the œsophagus to the rectum.

By Mr. Hastings, and Mr. Alcock, of England, we are told that, united with such phenomena, they thought that the air-passages were even more affected, everywhere throughout their whole extent, meeting with inflammation, and its immediate or secondary consequences—extravasations, adventitious membranes, ulcerations, &c.

Cross, an English writer of reputation, also found what he considered imperfect pustules, in the primæ viæ. "The rectum, the colon, and ilium," says he, "were marked with circular patches, distinct and white."

During the late prevalence of small-pox in this city, similar inspections were frequently made, many of which I had opportunities of witnessing. The phenomena, in general, corresponded very much to the preceding description; consisting of a mixture of inflammation and congestion, the one or the other state preponderating, according to the character of the case. But, though every part of the alimentary canal was occasionally observed to be intensely phlogosed, I saw nothing like regular pustules, nor did I hear of any such having been detected. The mucous tissue, however, of the bowels, in some instances, was found studded with prominent points, which were ascribed only to follicular inflammation, and, I believe, justly; having seen similar appearances in other diseases. It is, indeed, difficult to conceive how a pustule could be formed in this membrane, without its chorion were previously thickened by exposure to the air, such being its natural delicacy of texture, that it would probably burst, long before maturation; and in this opinion, I am confirmed by the report of Contunnius, Cazenave, and Schedel, who, in their numerous dissections, never saw pustules in this situation. It may hence, I think, be concluded, in conformity with general experience, that they are confined to the skin, exclusively, excepting in those rare instances of prolapsus of the uterus, or rectum, where, by long exposure to the atmosphere, the mucous is converted in the tegumentary tissue.

Concerning the exterior surface, the rete mucosum, in very slight affections, it is said, is only inflamed. But, almost invariably, the cutis vera becomes involved in the same state, ending in ulceration. The pock has, in the early stage, a cellular structure, the capsule of which is transparent, and secretes its lymphy contents; losing, at maturity, in a degree, this conformation, and becoming more of a sac, charged with pus. From the fifth to the eighth day of the eruption, may be seen a small circular slough of the cutis, which is held by some of the recent authorities, as the most unerring criterion of genuine small-pox, serving to discriminate it from all its affiliated affections. But it cannot uniformly be trusted. Cases of varicella I have repeatedly known productive of precisely the same effect. By this ulceration, the pits or scars are formed, which, when deep, indelibly remain.

It will not be difficult, after the foregoing exposition of the disease, to deduce its pathology. Like the exanthematous fevers, generally, it is radicated in the mucous membrane of the upper portion of the alimentary tube, to be thrown off, finally, on the tegumentary tissue, as the natural process of cure.

Of such an origin of small-pox, however, the evidence is stronger than of any other of these affections. Not to insist on the proofs of this position, which, in common with its congenera, it supplies, the early symptoms, the relief usually afforded by the appearance of the eruption, and the phenomena on dissection, it may be demonstrated in another way.

By repeated and well conducted experiments, it has been shown that the virus of small-pox cannot act on the skin, without an abraded or punctured cuticle, in which event it gives rise to local irritation, and, as an ultimate result, the inoculated disease.

The poison, then, must act on an interior surface. Elsewhere I have shown, and, I think, conclusively, that effluvia, contagious or otherwise, are not received through the lungs into the circulation. The stomach, therefore, being the remaining surface, to which the poison can have access, must constitute, of necessity, the seat of the initiatory impression.

It is on such an hypothesis, only, that the difference between the natural and the inoculated varieties of the disease is explicable.

Were the virus actually conveyed into the blood, and did it produce its effects, through the medium of that fluid, it would be of no moment at what point of the system the intromission took place. From the thorough commingling which the blood undergoes, in the route of the circulation, any portion of it being vitiated, the whole must be equally so, and the disease ensuing, in every case, be essentially the same.

Granting, however, as is certainly the fact, that the virus, instead of entering the blood, deranges the system through the medium of sympathy, and the difficulty of explanation at once vanishes.

Disease is violent, or the reverse, according to the importance of the organ injured, the nature of the lesion, and the power and extent of its connexions. No organ is more important than the stomach; the injury it suffers, in this case, is usually severe, and its sympathies, in strength and sensation, seem paramount to those of any other. Consequently, on its reception of the virus, it being deranged, casual small-pox, marked with severe symptoms, is the ordinary result.

But, under inoculation, the poison operating on a small portion of the skin, only, which is more capable of sustaining the injury, and the sympathies of that portion being comparatively weak and limited, it follows that the affection excited must be comparatively moderate. We see this difference strikingly illustrated in the endermic application of active remedies, where, to attain the effect of a dose by the stomach, three or four times the amount is required! What, in fine, is the lesion, that would not prove more serious, inflicted on the stomach, than the skin?

Nor, without the adoption of this hypothesis, can an explanation be had of some other phenomena of the disease. Casual small-pox, we have seen, shows itself on the fourteenth day, while that from inoculation, in half the time; and if, in each instance, the virus acts by entering the circulation, and commingling with the blood, how could this wide difference happen?

It further appears that it is of no moment, in the process of inoculation, whether a large or small quantity of the virus be inserted, the subsequent effect being not at all controlled by this circumstance. An ounce of the virus would, probably, not make a stronger constitutional impression than a single particle. Conceding this, which, to a certain extent, has been demonstrated, and it is scarcely possible to have more conclusive proof that the disease is propagated by irritation, and not by any contamination of the blood. Not a tittle of evidence, indeed, is there, that the blood is at all vitiated. Can we infect from it, which we ought surely to do, did it contain the virus? As in all other instances of disease, there is here, I repeat, a point primarily irritated, with which the system being brought into concert, a general disturbance ensues.

That such is true, as regards the inoculated disease, or, in other words, that all the subsequent effects are dependent on the original irritation, is as manifest, as that a stream of water, with its various ramifications, proceeds from a fountain by which they are fed. If this be not so, why are we vigilant to preserve the integrity of the parent pustule against those molestations by which it might be affected? Do we not know, that when this is disturbed in its progress, so as to subvert its specific action, that no specific impression is made on the system, and the act of inoculation becomes impaired, or is completely defeated? Duly estimating this fact, I think, no more will be exacted, to establish the position for which I am contending.

But, opposed to these views, the occasional occurrence of the disease in the fœtus, is commonly adduced, as of a very decisive nature. Directly the reverse, however, does it import. No one point in physiology is better established, or more generally allowed, than that there is no direct vascular connection between the parent and the offspring. Destitute, then, of any intercommunication by blood-vessels, how can the virus in the natural blood reach the fœtus? The basis of the hypothesis thus proved to be unsound, it of course falls. Two modes, explanatory of the infection of the fœtus, under these circumstances, may be suggested. The contagion might possibly, as has been suggested, though I do not believe it, penetrate through the vagina of the mother, and thus be brought to act on the uterine contents. There is no necessity, however, to resort to such a supposition. To a certain extent, the fœtus is an integral portion of the maternal system, evolved, sustained, and perfected by its resources, and susceptible of derivative impressions from it. Of the precise means, or manner of the connection between them, we are not informed. Nerves are said recently to have been demonstrated in the placenta and umbilical cord. But I insist on no such medium of connection, it not being essential to the support of my proposition. Though to be traced generally to an inosculation of nerves, numerous are the sympathies seemingly independent of it. Not to cite examples, superfluously, to this purport, what, I demand, is the nervous communication between the parotid gland and the testes, or the mammæ? What that between the uterus and mammæ? Let, however, the explanation be as it may, the fact is undeniable. It is by the adoption of sympathy, that a solution of this problem can only be had, and by which we are supplied with a clue, like that of Ariadne, to conduct us through the mysteries of the animal economy, otherwise more dark and intricate than the Dædalian labarynth, itself.

An additional remark or two, have I only to make, in regard to the pathology of small-pox. It sufficiently appears, from what has been said, that irritation commences in the mucous membrane of the stomach, particularly, which, in the natural course of things, is translated to the skin, as a part more capable of bearing it. The metastasis being complete, and firmly established there, the issue is generally favourable. But when the eruption is very extensive, and, especially, confluent, the functions of that integument, so important in the operations of the animal economy, are interrupted, its vitality impaired, or, perhaps, destroyed, and death ensues, from this condition chiefly or entirely.

Not the least curious circumstance connected with the history of this and its kindred affections, is the usual destruction of susceptibility of the system to any repetition of attacks. Great ingenuity has been exercised in the explanation of the enigma, without, however, a ray of light having been shed on it. It were idle to recite the vain conjectures of others, or to obtrude one of my own on the subject, and both I shall hence decline. An inquisitive personage once said to a venerable medical friend of mine, why cannot an individual have the small-pox twice? Tell me, he quickly replied, the reason he has it at all, and I will then endeavour to solve your difficulty! This smart retort, I suspect, conveys about as good an answer, though no explanation, as will be found in any of the graver speculations regarding this mystery.

We now come to the treatment of small-pox, which must be deemed an incurable disease, so far, at least, as that, in common with most, or, perhaps, all affections dependent on a specific contagion, it will run a definite course, in spite of our efforts. Nature, here works to the deliverance of the system, by a series of unintelligible processes which eventuate in the re-creation of the same sort of virus, as that which had originally excited the affection.

Disease is one of the curses entailed on our fallen condition, and, to perpetuate this particular class, it would seem to be ordained, that, we should be debarred the power of counteracting the agency, by which the *seminal principle* is regenerated.

In this respect, the same pains are taken as in the preservation of some of the productions of the animal and vegetable kingdoms. As the seeds, for future regeneration, are there elaborated, so is the principle of contagion, by these contagious fevers.

Could these cases be cured, a chasm would be made in the order of a particular design, which is not permitted. Yet, in conformity with the general benevolence of Providence, what cannot be entirely relieved, we are enabled to palliate, and such is the amount of our best endeavours in small-pox.

It may be collected, from the preceding views, that, where the disease is mild, and pursuing its career undisturbed by anomalous or exasperated affections, it were better to forbear the use of remedies, and to leave it pretty much to the efforts of nature, regulating mainly the diets and temperature. But, our aid being demanded, we adopt essentially the same plan in the variolous, as in ordinary fevers, accommodated to the condition, inflammatory, congestive, or mixed.

To the first of these states I am now to address myself. For the most part, it will be right to commence the treatment with an emetic, and, especially, where we have reason to suspect irritating ingesta, or an otherwise vitiated stomach. But it should be restricted to the earliest stage, and resorted to with all those cautions, regarding the existence of gastric inflammation, on the importance of which I have so often insisted. This remedy, thus timely exhibited, has a signally useful effect in the whole of the eruptive fevers, operating, probably, as well by rectification of the stomach itself, as by occasioning a determination to the cutaneous surface, thus preparing it for the reception of the eruption.

Next, a recurrence is to be had to the saline laxatives, so as to keep the bowels in a soluble state. Calomel was here greatly preferred by the older practitioners, under an impression, that it exercised some specific influence. But such a notion, I think, is unfounded, and, though not pernicious in moderation, mercurial purging is here unnecessay.

Excessive intestinal evacuation, particularly by drastic articles, should, indeed, be carefully avoided, as concentrating and fixing the disease in its original position, or tending greatly to the prevention of the translation of it to the skin, and in other respects, interfering with the natural process of cure.

Mild diaphoretics, the acetate of ammonia, or dulcified spirits of nitre, or a weak solution of emetic tartar, or the neutral mixture, or both combined, are very useful.

It has been remarked, that a perspirable condition is among the most salutary occurrences in the disease, and hence, the importance of the means by which it is excited or maintained.

The preceding remedies may answer. But, when the fever is high, with a heated skin, and much local determination, venesection cannot be dispensed with, and ought to be repeated, as the necessity of the case imposes. There is, however, some difference of opinion among practitioners, as to venesection; though the weight of authority is decidedly in favour of it. Commencing with Rhazes, we may trace the strongest recommendation of it, through Sydenham, Mead, Friend, Huxham, down to Armstrong, and still more recent writers, including, also, the best practitioners of our own country, at all times. That, however, it has no decided curative effect, must be admitted, and the same may be alleged of all other means. But though it does not arrest or change, it abates the intensity of action, and it is on this principle we resort to it, in common with the rest of the measures usually prescribed in the eruptive fevers, among which topical bleeding is of the utmost importance. Most of the vital organs are now deeply implicated, and to preserve the integrity of their structure, should be our principal aim. To relieve tenderness of the epigastrium, and the attendant gastric distress, or phlogosis of the brain, or lungs, or over-fulness of these organs, leeches or cups are incomparably effectual, and ought never to be neglected.

When the surface is very hot, applications of cold water have been recommended, even by aspersion. But, sanctioned as it is, by some high authority, the propriety of the practice is questionable. It were better, merely to sponge the surface, the utility of which cannot be too strongly urged.

Convulsions in children, which I have said are apt to occur, may, when slight, be relieved by cold applications to the head, and a stimulating pediluvium at the same time. But, should they be violent, the loss of blood, sinapisms to the extremities, and an opiate, become necessary.

The extent of the eruption, and other affections, being pretty nearly proportioned to the degree of reaction, the leading object is to restrain, and keep this down, with which intention the antiphlogistic plan must be pursued in every respect. But, above all, is it required, to have the patient in a cool and well ventilated apartment, more so than in any other disease, and to let him sleep on a mattrass, with little covering. It has, indeed, been found beneficial, in hot weather, to expose him, even to the cool external air, where an unusual degree of heat and fever prevails.

This cooling or antiphlogistic practice was adopted early in the history of the disease.

By Rhazes, such is, at least, recommended, which, in the revolutions of the science, and, especially, when the chemists and humoralists got possession of the schools, was entirely changed by their preposterous pathological and therapeutic views.

Considering the virus to be in the blood, and that it was to be expelled through the skin, by the force of fever, they adopted the most heating and alexipharmic measures to accomplish this end.

Giving an account of the practice of the time, Sennertus, who wrote at the beginning of the seventeenth century, says: "That, while using these means, every attention is to be paid, especially in winter, to the exclusion of cold air. The patient, therefore, is to be tended in a warm chamber, and carefully covered up, lest by closing the pores of the skin, the efforts of nature should be impeded, the humours driven upon internal organs, and matter, which ought to be expelled, retained within the body, to the imminent danger of the patient, and the certainty of increasing restlessness, fever, and other symptoms."*

As might be supposed, the mortality, from such a plan, was immense. The keen sagacity of Sydenham discovered the error, and dictated the appropriate reformation, in the revival of the ancient practice, which has since been established by lengthened and concurrent experience.

(To be continued.)

---

*Radical Cure of Prolapsus Uteri.*—M. Velpeau recently performed the following operation in an old case of prolapsus of the womb, complicated with a cystocele.

Pinching together the mucous coat of the vagina, he cut away three slips of it, one from the anterior part, and the other two from the sides of the canal. Each of the slips was nearly an inch wide, and two inches and a half long. M. Velpeau had previously inserted the ligatures, so that there was no difficulty experienced in bringing the edges of the wound together. Three months had elapsed when the report was made, and the operation had been successful.

* De Variolis et Morbillis: Tom. vi.

